# In-vitro influence of the use of an erythritol powder through air polishing on the surface roughness and abrasiveness of various restorative materials

**DOI:** 10.1371/journal.pone.0270938

**Published:** 2022-07-07

**Authors:** David Reinhart, Preeti Singh-Hüsgen, Stefan Zimmer, Mozhgan Bizhang

**Affiliations:** Department of Operative and Preventive Dentistry, Faculty of Health, Witten/Herdecke University, Witten, Germany; Klinikum der Johann Wolfgang Goethe-Universitat Frankfurt Klinik fur Nuklearmedizin, GERMANY

## Abstract

The aim of this in-vitro study is to compare the prophylaxis powder Airflow^®^ Plus to a conventional prophylaxis paste with regards to surface abrasion and roughness on four different restorative materials. A total of 80 samples were fabricated, including 20 of each investigated material. Among those were a nanocomposite (Ceram X Spectra^™^ ST, Dentsply), a glass ionomer cement (Ketac Fill^™^, 3M^™^), a cast metal alloy (Bio Maingold SG^®^, Heraeus Kulzer) and a ceramic (HeraCeram^®^ Saphir, Heraeus Kulzer). Of each material, all samples were equally divided into two groups. Samples in one group were treated with AirFlow^®^ Plus using the AirFlow^®^ Prophylaxis Master (EMS, Switzerland) (Group AF) and the ones in the other group with Prophy Paste (Cleanic^™^, Kerr, Austria) (Group CL) on a rubber cup. Applied force amounted to 1.5 N at 2000 rpm. Under controlled reproduceable conditions, a 10-year interval with 4 application per year, a total of 200 seconds, was simulated. Size of each sample amounted to 6 mm in diameter and 2 mm in height. Half side of each sample were treated. While comparing the treated and untreated area of each sample, surface abrasion and roughness were measured using an optical 3D system. Roughness was measured based on the arithmetic roughness average of the surface (Ra) and root mean square of the surface roughness (Rq). The statistical evaluation of the data was carried out using the non-parametric Mann-Whitney-U-test, Wilcoxon-test and the Kruskal-Wallis test for group comparisons. In conclusion, the use of the rubber cup with Prophy Paste caused a significantly higher abrasion on composite, ceramic and gold compared to the AirFlow^®^ Plus powder (p < 0.05). In group AF, the significant highest values for Ra were determined on GIC, followed by composite, gold and then ceramic in intragroup comparison. Ra on GIC was significantly higher in group AF (p < 0.05).

## Introduction

Dental prophylaxis and periodontal maintenance therapy have been proven important in achieving good oral health [1]. It is known that gingival inflammation is an outcome of an unsatisfactory supragingival plaque control [2]. The interval between treatments and the patient’s-motivation depends on caries risk, age, the state of periodontal disease and systemic and environmental factors [3]. In addition to the conventional method of using polishing paste with a rubber cup or brush, serval ways of removing dental plaque and extrinsic stains exist. One such method is the mix of air-abrasive powder and water (air-polishing). Depending on caries risk management or the criteria for supportive periodontal treatment, the indication of prophylactic treatment in form of air polishing can conclude in 4–6 times a year [3, 4] Various studies have documented the use of air-polishing compared to rubber cup, hand and ultrasonic scalers, and proven its effectiveness and ease of usage [5–8].

The early air-polishing devices use sodium bicarbonate (NaHCO_3_) powder with a particle size of up to 250 μm [7, 8]. However, this highly abrasive powder, can cause potential damage to soft and hard tissue as well as restorative materials [7–10]. At the same time, it has been shown that the use of sodium bicarbonate may cause abrasion and dulling effects on restorative materials, such as cast metal alloy, composite and glass-ionomer, which leads to material loss over time [11, 12] and results in an increased surface roughness due to abrasion. A surge in surface roughness causes an increase in the bacterial adhesion and therefore gingival inflammation and secondary caries [13, 14]. Due to those harmful side- effects of abrasive powders and instruments a search for lesser abrasive powders for dental prophylaxis and maintenance therapy was initiated. With this purpose in mind, a range of powders consisting of calcium carbonate, aluminium trihydroxide, glycine and erythritol were developed. While usage of calcium carbonate limited due the modest water solubility, Johnson et al. concluded that aluminium trihydroxide should be avoided on resin composites, resin-modified composites, glass ionomer cements and the margins of cemented restorations due to its abrasiveness [7, 10, 15]. Glycine is a naturally occurring amino acid with a smaller particle size than NaHCO_3_ [7, 16]. Erythritol is a sugar alcohol with an even smaller average particle size of 14 μm. Therefore, glycine- and erythritol-based powders produce significantly less surface damages compared to other abrasive powders, contrasted with NaHCO_3_ [14, 17, 18]. Besides the powder characteristics i.e. particle size or shape, the instrument settings affect the outcome of abrasion. A higher amount of pressure, waterflow, a shorter distance and time result in higher defect depths, while a lower angulation of the nozzle leads to lower defect depth [19, 20]. In dental prophylaxis, air polishing is used for eliminating plaque and stains more effectively compared to conventional instrumentation, like the use of a rubber cup with prophylaxis paste [7]. Earlier use of air polishing was limited to supragingival surfaces. Nevertheless, the effective application of erythritol in subgingival areas has been demonstrated in various studies and proven the benefits in supportive periodontal therapy [21–23]. Moreover, the frequently use of air-polishing is a proven method in prophylactic maintenance or supportive periodontal therapy. Hence it is important to minimize the caused side effects as abrasion and increased roughness [8]. The objective of this in-vitro study was to investigate an erythritol powder (AirFlow^®^ Plus, EMS) in comparison to the conventional method of using a rubber cup and pumice paste with regards to the abrasion and roughness caused on restorative materials like composite, a cast metal alloy, ceramic and glass ionomer cement. The null hypotheses state that (1) the abrasion caused by AirFlow^®^ Plus is not significantly higher compared to Cleanic^™^ prophy-paste and, thus (2) the roughness of the restorative surfaces will not be significant higher, following the use of AirFlow^®^ Plus in comparison to Cleanic^™^ Prophy-Paste. Additionally, the surface abrasion (3) and the roughness (4) on permanent materials is not higher after the use of AirFlow^®^ Plus compared to a non-permanent restorative material. The aim of this study was to evaluate the surface abrasion and roughness behaviour of erythritol powder on four dental restorative materials following simulation for a period of ten years.

## Material and methods

The G*Power software (G*Power; University of Dusseldorf, Dusseldorf, Germany) was used to calculate the sample size [24]. Variables used for sample size calculation were that of abrasion between AirFlow^®^ Plus and Cleanic^™^ Prophy-Paste. The level of significance was set at 0.05, power of the study (1-ß) was amounted to 0.8 and the effect size was 1.2, which resulted in a sample size of n = 10 per material for each treatment group. A total of 80 samples were fabricated by placing 20 test specimens of each material i.e. composite (nanocomposite, Ceram X Spectra ^™^ ST, Dentsply), gold (cast alloy with high gold content, Maingold SG^®^–Heraeus), ceramic (veneering silicate ceramic, HeraCeram^®^ Saphir–Heraeus) and a glass ionomer cement (GIC; Ketac Fil^™^– 3M ESPE) on plaster cubes. GIC was used as a negative testing group, as it is a temporary restorative material with little long-term oral stability. All samples received a surface treatment, so that a relatively uniform initial situation was established. Therefore, each sample underwent a parallelization and uniform finishing procedure due multi-stage wet grinding process (Exakt, Norderstedt, Deutschland) with grit sizes from 500–4000. Following this, each sample was divided in two parts, the reference area and treatment area were divided equally with a single scalpel cut. The cutting line was not considered in the analysis. The reference area was covered with a tape and a protection shield, a thin aluminum alloy plate which was fixed on it. The 20 samples of each restorative material were equally divided into two groups of 10 each. Each group was either treated with a powder-water polishing device or polishing paste using a rubber cup on a handpiece:

1. Group AF: AirFlow^®^ Plus—erythritol powder (EMS SA, Switzerland) (Table 1).
2. Group CL: Cleanic^®^ prophy-paste (Kerr, Switzerland) (Table 2).

**Table 1:** Standardized settings for group AF.

Group AF	
Working angle	45°
Working Distance	3 mm
Water flow rate (Airflow)	Setting 10
Air pressure (Airflow)	Setting 10–3.1 bar
Powder	Airflow^®^ powder plus (Erythrit, Chlorhexidindiacetat)
Particle size	14 μm
Working time each sample	200 s

Detailed information about abrasive powder, fixed setting and spacing

**Table 2:** Standardized settings for group CL.

Group CL	
Working angle	90°
Handpiece transmission	1:1
Rotational speed	2000 rpm
Applied force	1.5 N
Paste	Cleanic Prophy (Ethanol, Natriumfluorid, Titandioxid, Glycerin)
RDA / REA	27 / 3.4
Amount	0.05 g each 15 s
Cup	ProCup Hard (Kerr)
Working time each sample	200 s

Detailed information about prophylactic paste and fixed setting

rpm: rounds per minute, RDA: radioactive dentine Abrasion, REA: radioactive enamel abrasion

**Fig 1:**
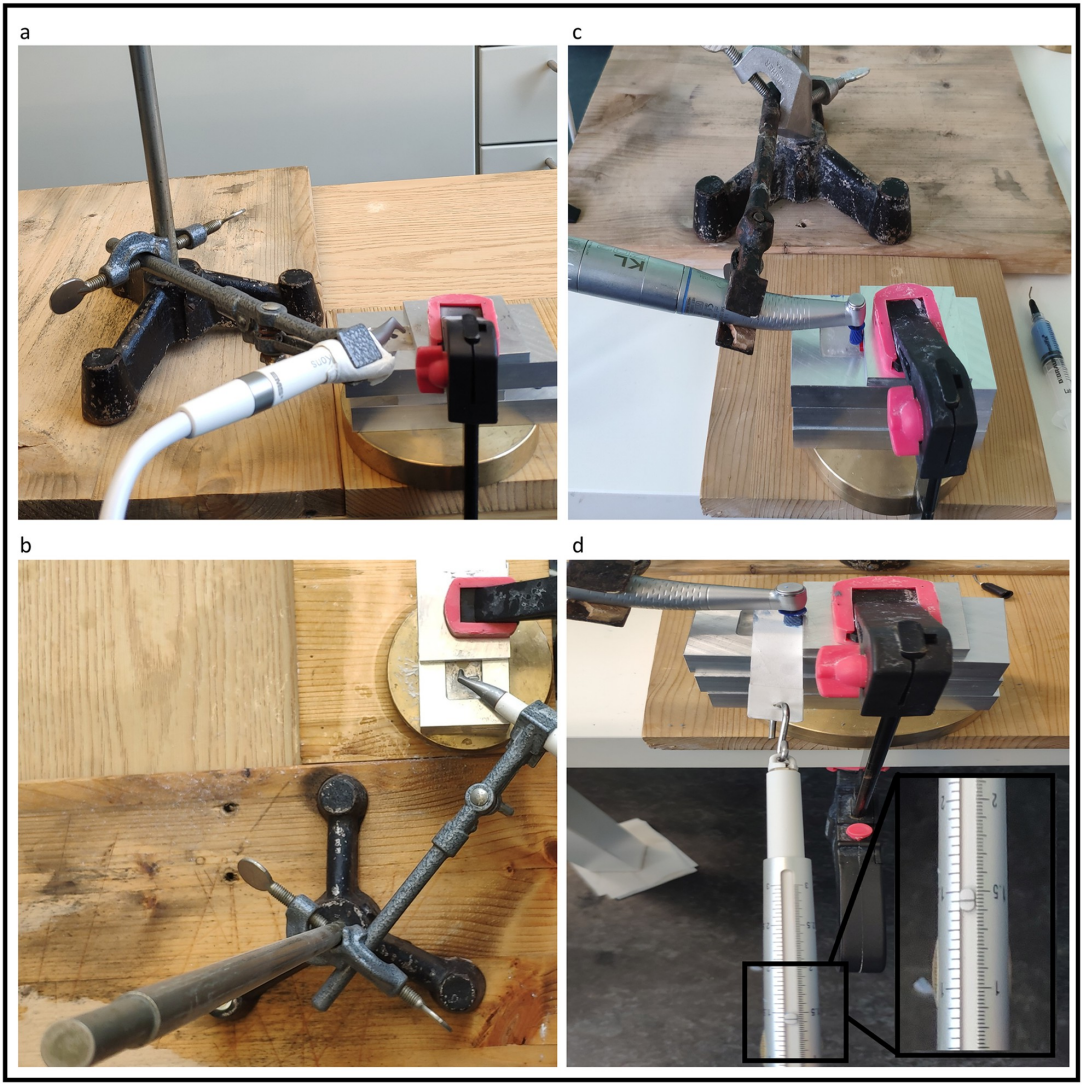
Constructions. Side and top view of the device construction for group AF (a-b) plus side and top view of the device construction for group CL (c-d).

To simulate a 10-year interval of prophylactic maintenance a treatment duration of 200 s was set. Based on the established time for using prophylactic paste (approx. 5 s during one session), the appliance of abrasive powder was adapted for comprehension [25]. Group AF was treated with the powder-water jet device (AirFlow^®^ Prophylaxis Master, EMS SA, Switzerland) and operated with maximum air pressure and maximum water flowrate. One operator carried out rotating movements around the rotating axis of the specimen. The test samples were instrumented under standardized and reproducible conditions from a distance of 3 mm and at an angle of 45°. The water and powder tanks were filled to the maximum at intervals of 100 s. Similarly, the treatment of the group CL samples was carried out under standardized conditions. The same operator used the rubber cup with rotating motions at a working angle of 90° for 200 s and applied a force of 1.5 N, set with a spring balance. Prophylaxis paste of 0.05 g was inserted into the rubber cup every 15 s and the applied force was controlled with the spring scale. A stopwatch was used to keep track of time. The reference area remained covered throughout the treatment procedure. Stable rotating movements of the devices were made possible by attaching the device construction to a stand. The constructions for both groups can be seen in Fig 1. Following treatment, the surface abrasion and roughness of all samples was measured with an optical 3D measuring system (InfiniteFocus–Alicona^®^ Imaging GmbH, Austria). The focus variation, recorded in the current EN ISO standard 25178 on the surface roughness measurement, is the core technology of the Alicona measuring systems. A setting for 10x and 20x magnification was used respectively to evaluate the abrasion and roughness. Photographs were taken to ensure the parameters had a size of 1020 μm x 815 μm. Each photograph showed an equal part of the treated and untreated area. For orientation, the scalpel section on the one hand and the processing area on the other hand were taken. In order to measure the abrasion, the average difference in height of the treated and untreated area was calculated. Simultaneously, the roughness was evaluated by measuring the difference between the treated and untreated area. The following parameters according to EN ISO 25178 were used to describe the material roughness [26].

Ra—arithmetic roughness average of the surface
Rq—root mean square of the surface roughness

The depth image was aligned based on the untreated side of each sample. Thus, it was virtually aligned as the initial x-coordinate. Furthermore, the roughness was compared to the primary untreated area. The difference in roughness for Ra and Rq between treated and untreated area was calculated. Three values were noted for each specimen to evaluate the abrasion. In order to determine the roughness, six values were determined for each parameter of Ra and Rq. Afterwards, the parameters were compared in the application group itself, to show differences between the restorative materials. Furthermore, the two different groups, AF and CL, were compared and the outcomes evaluated.

### Statistical analysis

IBM SPSS Statistics 26 was used for statistical analysis. The Kolmogorov-Smirnov test showed an inhomogeneous distribution of data. Due to an asymmetric distribution of data, the non-parametric Kruskal-Wallis test and Mann-Whitney-U test were used for analysis. The significance level was set at p < 0.05. Parameters for abrasion and roughness were compared between the reference area and treatment area in each group AF and CL. Using Bonferroni correction, the p-value was set to 0.01 in order to reduce the risk exposure in the respective group comparison.

## Results

Before statically evaluating the results, the mean values were calculated. For abrasion, the average of three values from different recordings were determined. The same was done for the roughness values Ra and Rq, with six values and recordings. The inter-group comparison was carried out with a p-value of 0.05 and for intra-group comparisons a p-value of 0.01 was selected. After Bonferroni correction, the probability of error *p* and the level of significance were defined as *p* = 0.01. Table 3 shows the results for abrasion, while Table 4 shows the p-values of the intragroup comparison for abrasion. During the comparison of the two different application methods AF and CL, the highest material loss was determined for both methods for GIC. In group AF a median of 1.25 μm for abrasion on gold was emitted, while in group CL it amounted for 7.14 μm. There was no significant difference (p < 0.05) between the two application forms for the abrasion of GIC with a median of 27.19 μm in group AF and 25.32 μm. For composite, gold and ceramic a significantly lower abrasion was determined in group AF compared to CL (p < 0.05). In group AF there was a significantly higher material loss in the form of abrasion for GIC compared to composite, gold and ceramic. The abrasion on ceramic was statistically lower (p < 0.01) compared to the other materials in group AF and CL. On composite the abrasion was significantly higher compared to gold (p < 0.01) in both groups.

**Table 3:** Median, maximum, minimum, first and third percentile of abrasion of the different materials in micrometres and mean loss of material after treatment with AF and CL.

Variable	Group	Material						n	p-value*
			Median	Min	Max	Q1	Q3		
Abrasion [μm]	AF	Composite	10.76 ^a^	9.06	11.98	9.44	11.48	10	< 0.001*
		GIC	27.19 ^b^	24.34	30.28	25.95	28.71	10	= 0.143
		Ceramic	0.72 ^c^	0.69	0.77	0.72	0.74	10	< 0.001*
		Gold	1.25 ^d^	1.17	1.38	1.22	1.30	10	< 0.001*
	CL	Composite	18.79 ^a^	15.75	22.41	18.21	20.38	10	< 0.001*
		GIC	25.32 ^b^	19.63	28.22	24.85	27.14	10	= 0.143
		Ceramic	2.50 ^c^	1.78	3.00	2.23	2.75	10	< 0.001*
		Gold	7.14 ^d^	5.76	8.28	6.31	7.73	10	< 0.001*

* Differences between test groups were statistically significant in the intergroup comparison p < 0.05 by Mann-Whitney-U-test.

Superscript letters indicate the statistically significant differences between the same treatment groups (p < 0.01 by Kruskal-Wallis and Mann-Whitney-U-test)

Min: minimum; Max: maximum; Q1: 25% Percentile; Q3: 75% Percentile; n: sample number; GIC: glass ionomer cement.

**Table 4:** Intragroup comparison of p-values between the median abrasion of used restoration materials*.

Group	Composite		GIC		Ceramic		Material
	AF	CL	AF	CL	AF	CL	
p-value*	< 0.001	< 0.001					GIC
	< 0.001	< 0.001	< 0.001	< 0.001			Ceramic
	< 0.001	< 0.001	< 0.001	< 0.001	< 0.001	< 0.001	Gold

* P-values calculated by Kruskal-Wallis and Mann-Whitney-U-test, adjusted p-value due Bonferroni correction (p < 0.01); GIC: glass ionomer cement

Whilst Table 5 shows the results of the roughness parameters Ra and Rq, Tables 6 and 7 show the p-values for Ra and Rq for intragroup comparison. These parameters were found to have lower values in group AF compared to group CL, except on GIC. On composite a statistically significant lower roughness (p < 0.05) was found and also on gold and ceramic. On GIC a significantly higher roughness (p < 0.05) was found for Ra and Rq in group AF than in group CL.

**Table 5:** Median, maximum, minimum, first and third percentile for Ra and Rq of the different materials in nanometres. Roughness of materials increases after treatment with AF and CL.

Variable	Group	Material						n	p-value*
			Median	Min	Max	Q1	Q3		
Ra [nm]	AF	Composite	3.86 * ^a^	3.43	4.21	3.7	4.12	10	< 0.001*
		GIC	140.6 ^b^	127.67	156.39	137.73	148.23	10	< 0.001*
		Ceramic	2.04 * ^c^	1.66	2.30	1.96	2.28	10	< 0.001*
		Gold	3.22 * ^d^	2.57	4.01	3.12	3.38	10	< 0.001*
	CL	Composite	10.54 ^a^	8.81	12.04	9.79	11.45	10	< 0.001*
		GIC	47.06 * ^b^	42.00	57.24	42.52	50.20	10	< 0.001*
		Ceramic	5.52 ^c^	4.64	6.41	5.05	5.87	10	< 0.001*
		Gold	13.90 ^d^	12.01	16.86	13.53	16.10	10	< 0.001*
Rq [nm]	AF	Composite	5.12 * ^a^	4.31	5.7	4.82	5.32	10	< 0.001*
		GIC	161.01 ^b^	146.91	175.69	154.75	165.72	10	< 0.001*
		Ceramic	4.19 * ^c^	3.76	5.10	4.13	4.57	10	< 0.001*
		Gold	5.00 * ^a^	4.39	5.42	4.75	5.07	10	< 0.001*
	CL	Composite	15.62 ^a^	12.93	18.95	13.86	16.40	10	< 0.001*
		GIC	62.61 * ^b^	56.41	74.69	59.73	66.39	10	< 0.001*
		Ceramic	10.35 ^c^	9.07	11.13	9.90	10.54	10	< 0.001*
		Gold	16.68 ^a^	14.72	22.32	15.45	20.14	10	< 0.001*

* Differences between test groups were statistically significant in the intergroup comparison p < 0.05 by Mann-Whitney-U-test. Marked values were statistically significant lower.

Superscript letters indicate the statistically significant differences between the same treatment groups (p < 0.01 by Kruskal-Wallis and Mann-Whitney-U-test)

Min: minimum; Max: maximum; Q1: 25% Percentile; Q3: 75% Percentile; n: sample number; GIC: glass ionomer cement.

**Table 6:** Intragroup comparison of p-values between the median Ra of used restoration materials*.

Group	Composite		GIC		Ceramic		Material
	AF	CL	AF	CL	AF	CL	
p-value*	< 0.001	< 0.001					GIC
	< 0.001	< 0.001	< 0.001	< 0.001			Ceramic
	0.002	< 0.001	< 0.001	< 0.001	< 0.001	< 0.001	Gold

* P-values calculated by Kruskal-Wallis and Mann-Whitney-U-test, adjusted p-value due Bonferroni correction (p < 0.01); GIC: glass ionomer cement

**Table 7:** Intragroup comparison of p-values between the median Rq of used restoration materials*.

Group	Composite		GIC		Ceramic		Material
	AF	CL	AF	CL	AF	CL	
p-value*	< 0.001	< 0.001					GIC
	< 0.001	< 0.001	< 0.001	< 0.001			Ceramic
	0.315	0.089	< 0.001	< 0.001	0.002	< 0.001	Gold

* P-values calculated by Kruskal-Wallis and Mann-Whitney-U-test, adjusted p-value due Bonferroni correction (p < 0.01); GIC: glass ionomer cement

The least statistically significant increase in roughness for Ra and Rq was achieved in group AF on ceramic and the highest statistically significant increase in roughness was reported in group AF on GIC. The parameter Ra showed a significantly higher increase in roughness (p < 0.01) on composite contrasted to the metal cast alloy. For the parameter Rq the order was similar, but no statistically significant difference (p < 0.01) in roughness was found between composite and gold. Similarly, in group CL the least statistically significant increase in roughness for the parameter’s Ra and Rq was achieved on ceramic (p < 0.01), while the highest was found on GIC. For Ra a statistically significantly higher increase (p < 0.01) in roughness for gold compared to composite was achieved. Rq results in group CL were analogous to group AF.

**Fig 2:**
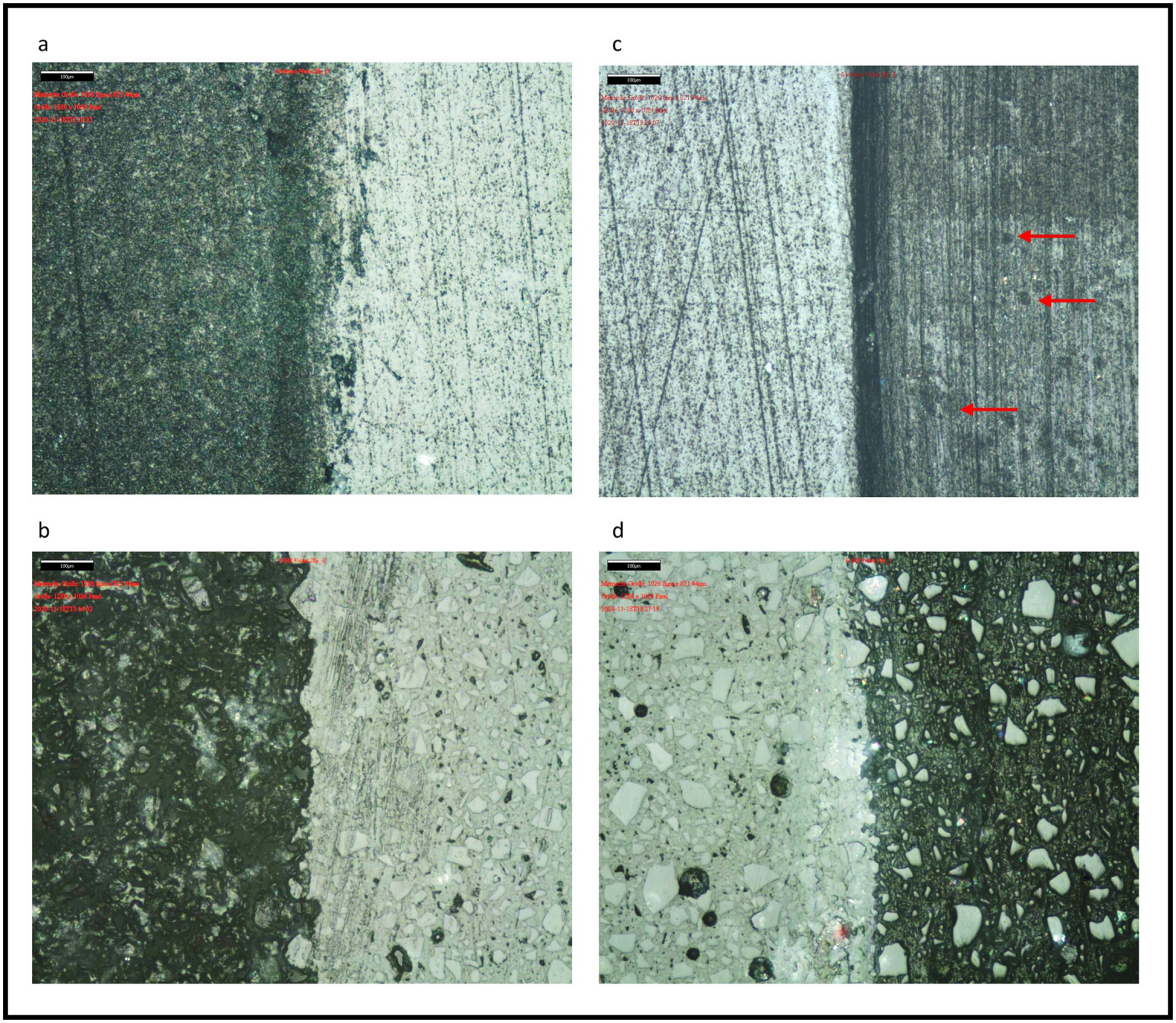
Probes following air polishing (a-b) and following prophylaxis paste (c-d). a–composite (left treated, right untreated); b–GIC (left treated, right untreated);c–composite (left untreated, right treated); d–GIC (left untreated, right treated; Red arrows mark pitting after treatment with prophylactic paste. GIC: glass ionomer cement.

**Fig 3:**
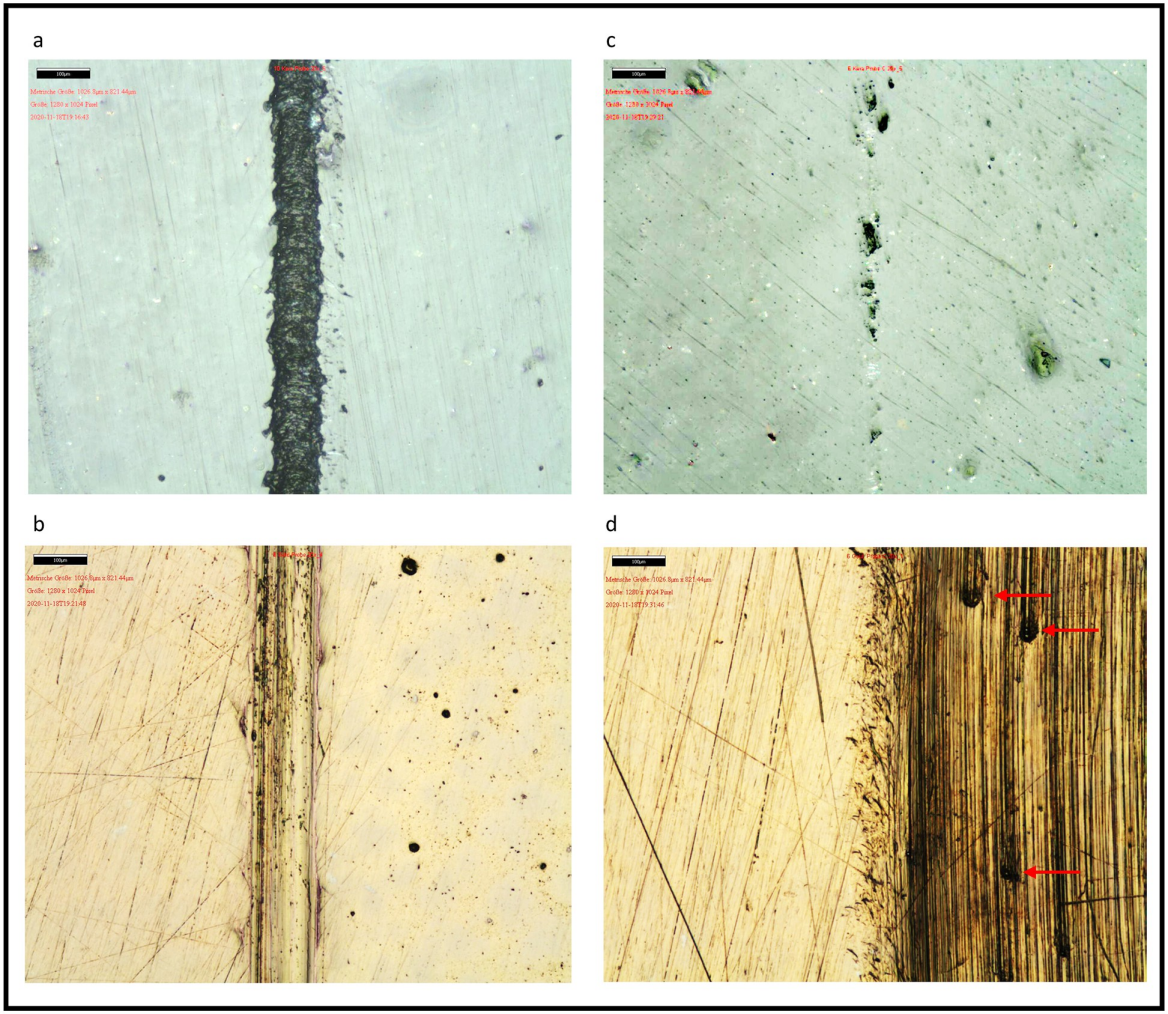
Probes following air polishing (a-b) and following prophylaxis paste (c-d). a–ceramic (left untreated, right treated); b–gold (left untreated, right treated); c–ceramic (left untreated, right treated); d–gold (left untreated, right treated); Red arrows mark pitting after treatment with prophylactic paste.

Figs 2 and 3 show the magnification of composite, GIC, ceramic and gold samples for reference and treatment areas. The marked dividing line between the areas has not been considered for the evaluation.

Statistical analysis with the Mann-Whitney-U-test (p < 0.05) for the comparison of the two application methods revealed the following:

- Abrasion on composite, ceramic and gold was significantly lower in group AF
- Abrasion on GIC showed no significant difference between the AF and CL groups
- Ra and Rq was significant higher in group CL on composite, gold and ceramic

Comparison using the Mann-Whitney-U-test (p < 0.01) within the groups AF and CL, revealed the following:

- The order from higher to lower abrasion in both groups performed as follows: GIC > composite > gold > ceramic
- Ra in group AF was significantly higher on composite compared to gold, while the values for Ra and Rq were the highest for GIC and lowest for ceramic
- In group CL Ra on gold was significantly higher compared to composite
- Rq for gold and composite in both groups AF and CL showed no statistically significant difference

## Discussion

This study aims was to evaluate the benefit of using an erythritol powder, AirFlow^®^ Plus for prophylaxis compared to the conventional method of using a rubber cup with a prophylaxis paste, Cleanic^™^ Prophy-Paste. In the present study, the surface abrasion and roughness of all samples was measured after treatment using a confocal microscope. This method has the advantage of objective quantitative 3D measuring of abrasion and roughness parameters from the probes without altering the surface.

Despite the fact that the treatment time for both methods was the same in the present study, other studies have already proven that stain and plaque removal is more efficient with an air powder system [7]. Several studies have further shown abrasive effects on restorative materials after air polishing, while comparing different powders used on different materials [14, 17, 18]. Various studies have demonstrated that both abrasive powders and polishing pastes result in comparable roughness parameters and abrasion values [16, 27]. As shown in Tables 3 and 5, the air polishing powder tested in the present study shows less abrasion and roughness on composite, ceramic and gold compared to the use of a rubber cup with a prophylaxis paste. The only increase on roughness after using the erythritol-based powder was shown on GIC, where for Ra the median of 140.6 nm compared to 47.06 nm in group CL resulted (Table 5). This can be attributed to the high wear properties and softer structure of GIC [28]. The softer structure leads additionally to an inhomogeneous abrasion of the surface with smaller and larger concavities, which attract more of the emission powder particles [29]. When a vertical working mechanism is applied the rotating rubber cup and a horizontal removal takes place, the already existing cavities of the GIC remain recessed until the surface has been evenly removed. The production of the samples themselves could have further led to different initial conditions. In addition, the water sorption while using AirFlow could be increased and lead to higher roughness compared to the prophylactic paste, where less water interacts with the surface [30]. Apart from these considerations, the chosen treatment interval is extremely long, which was chosen to illustrate the effect or side effect. Moreover, the recommended duration of glass ionomer cement as a temporary filling material is less than 1/10 of the simulation time.

A dynamic test sequence in the form of moving machining was chosen for this study, which aimed a wide-area treatment instead of a singular working point. While considering the different forms of operation without changing the parameters of distance and contact pressure, this study was carried out with a rotating axis of the experimental setup to be comparable to the in-vivo application. In addition, the experimental set-up was not changed during the entire experiment, as only the samples from the holders made for this purpose were exchanged. The surface of the nano-hybrid composite appears darker following both treatment forms, while the outcome in the macroscopic view shows a loss of glaze. However, the structure of composite following treatment with AirFlow^®^ Plus, appears to be more homogenic with a median for Ra of 3.86 nm, compared to the treatment with Cleanic prophy-paste and a resulted median of 10.54 nm. The structure of the treated surface shows grooves and little pits following the use of rubber cup and prophylaxis paste (Fig 1c). An analogous appearance can be seen on the gold sample, which explains the higher values of roughness for Ra (Fig 2d). While Ra on composite is significantly higher in group CL, Rq shows no significant difference. This may be caused by the fact that Ra values are less sensitive to single deep grooves or pits compared to Rq [31]. The parameter for roughness, especially Rq, shows in both application forms (AF and CL) no significant differences between gold and composite. This can be attributed to an improved wear resistance of nanocomposites, caused by agglomeration of the filler particles. Moreover, an improved bonding in the organic and inorganic hybrid layer may lead to less abrasion, which in turn leads to lower surface roughness [32]. It is known that the particle size and form of abrasive powders are related to their abrasiveness [8]. Different studies have shown that smaller particles like glycine or erythritol cause lesser damage to teeth or dental restorations [16, 17]. Thus, innovation of newer powders with tinier particles could lead to a better outcome in terms of abrasiveness and roughness compared to conventional methods such as a prophylaxis paste used with a rubber cup. On the other hand, this may lower the efficiency of the powder by increasing the time required for stain removal. Figs 2 and 3 demonstrate the abrasive effect of rubber cup usage as a flattening outcome shown by an even vertically abraded surface. This effect was found to be less homogenic on a softer restorative material like GIC. The effect of dynamic instrumentation on permanent restorations, like composites, cast metal alloys and ceramics creates a homogeneous surface abrasion with lesser surface damage. In this study, all samples were prepared in a similar manner to achieve a smooth and even surface. Therefore, further research must be carried out to investigate the effect of AirFlow^®^ Plus on rough and uneven surfaces. The fact that the samples underwent no artificial aging also limits the transfer to in-vivo circumstances. Furthermore, the limitation of this in-vitro study tried to mimic the device movements to simulate a real clinical situation. In-vitro studies examining dental abrasions offer a standardized setting that eliminates dentist related parameters such as variance in movements, force and time as well as environmental factors. In addition, this study simulated an application time of ten years. The fact that this study was carried out without the application of plaque substitutes further limits the results, because the aim of this study dealt with the measurement of abrasion and roughness instead of effectiveness. Therefore, we suggest that future studies include further parameters to simulate in situ prophylaxis treatment. Taking into consideration the abrasion and roughness performance that might be improved by a shorter prophylaxis treatment and patient compliance. Concurrently the studies should include different devices, powders and future dental materials.

## Conclusion

Within the limitations of this study, it has been found that treatment with an erythritol powder, AirFlow^®^ Plus leads to less abrasion and surface roughness of permanent restorative materials compared to conventional dental prophylaxis methods. The outcome of AirFlow^®^ Plus on temporary restauration material limit the application and requires further investigation in shorter simulations. Both methods of tooth cleaning and polishing produced maximum surface roughness on glass ionomer cement, followed by composite and gold, while the least rough surface was measured on ceramic. Despite this study being carried out in a laboratory setting, the results of the current study show that an erythritol powder, AirFlow^®^ Plus can be recommended for dental prophylaxis on permanent restauration, as far as further in-situ or in-vivo investigations support the assumption.

## Supporting information

S1 File Minimal data set. Raw data extracted from "Alicona imaging" (Alicona^®^ InfiniteFocus–Bruker Alicona, Graz, Austria) which were used for statistical analysis. Full data availability.
(XLSX)

S2 File Raw data incorporated into SPSS for statistical analysis. (SAV)

S3 File Statistical analysis in SPSS. Full data availability over statistical tests which were made through the data of S1 File.
(RAR)

S4 File Statistical analysis summed in pdf. Tables extracted from S3 File and summed up in pdf for better overview.
(PDF)
