# Supplementary material for: In-vitro influence of the use of an erythritol powder through air polishing on the surface roughness and abrasiveness of various restorative materials
Source: PLoS One. 2022 Jul 7;17(7):e0270938. doi: 10.1371/journal.pone.0270938 (PMC9262204; doi:10.1371/journal.pone.0270938)
Supplement: S4 File — Tables extracted from S3 File and summed up in pdf for better overview. (PDF) [file pone.0270938.s004.pdf]

Machine Translated by Google

Legend:

- Treatment AF	=	Treatment group AF (Air Flow)
- Treatment CL	=	Treatment group CL (prophylaxis paste Cleanic)
- KM	=	Composite
- Gold	=	gold
- GLZ	=	Glass ionomer cement (GIC)
- ceramics	=	Ceramic

Machine Translated by Google

Processed Casesa

	cases					
	Locked in		Locked out		All in all	
	N	percent	N	percent	N	percent
Difference_Ra_nm * Group_Maerial	40	100.0%	0	0.0%	40	100.0%
Difference_Rq_nm * Group_Maerial	40	100.0%	0	0.0%	40	100.0%
mean_removal_µm * Group_Maerial	40	100.0%	0	0.0%	40	100.0%

a. group_treatment = AF

Summary of Casesa

Group_Maerial		Difference_Ra_nm	Difference_Rq_nm	average_removal_µm
1	minimum	3.43	4:31	9.06
	maximum	4:21	5.70	11.98
	Average	3.87	5.06	10.55
	Std Deviation	,26	,41	1.09
2	minimum	127.67	146.91	24.34
	maximum	156.39	175.69	30.28
	Average	141.65	160.90	27.22
	Std Deviation	8.50	9:12	1.88
3	minimum	1.66	3.76	,69
	maximum	2.30	5.10	,77
	Average	2.05	4.30	,73
	Std Deviation	,21	,39	,02
4	minimum	2.57	4.39	1:17
	maximum	4.01	5.42	1.38
	Average	3.26	4.94	1.26
	Std Deviation	,41	,29	,06

a. group_treatment = AF

Machine Translated by Google

Processed Casesa

	cases					
	Locked in		Locked out		All in all	
	N	percent	N	percent	N	percent
Difference_Ra_nm * Group_Maerial	40	100.0%	0	0.0%	40	100.0%
Difference_Rq_nm * Group_Maerial	40	100.0%	0	0.0%	40	100.0%
mean_removal_µm * Group_Maerial	40	100.0%	0	0.0%	40	100.0%

a. group_treatment = CL

Summary of Casesa

Group_Maerial		Difference_Ra_nm	Difference_Rq_nm	average_removal_µm
1	minimum	8.81	12.93	15.75
	maximum	12.04	18.95	22:41
	Average	10.55	15.52	19.09
	Std Deviation	1.07	1.98	1.92
2	minimum	42.00	56.41	19.63
	maximum	57.24	74.69	28.22
	Average	47.55	63.36	25.43
	Std Deviation	5.24	5.29	2.37
3	minimum	4.64	9.07	1.78
	maximum	6.41	11:13	2.99
	Average	5.50	10.24	2.45
	Std Deviation	,53	,63	,39
4	minimum	12.01	14.72	5.76
	maximum	16.86	22.32	8.28
	Average	14.38	17.68	7.09
	Std Deviation	1.57	2.62	,84

a. group_treatment = CL

Machine Translated by Google

Kolmogorov-Smirnov test of conformity

		average_removal_ µm	Difference_Ra_n m	Difference_Rq_n m
N		10	10	10
parameters of normal distributionb,c	Average	10.5545	3.8706	5.0639
	Std Deviation	1.09236	,25804	,40636
Most extreme differences	Absolutely	,241	,117	,144
	positive	,178	.095	,124
	negative	-.241	-,117	-.144
Statistics for test		,241	,117	,144
Asymptotic Significance (2-sided)		,104d	,200d,e	,200d,e

a. group_treatment = AF, group_material = 1

b. The distribution to be tested is a normal distribution.

c. Calculated from the data.

i.e. Significance correction according to Lilliefors.

e. This is a lower limit of real significance.

Kolmogorov-Smirnov test of conformity

		average_removal_ µm	Difference_Ra_n m	Difference_Rq_n m
N		10	10	10
parameters of normal distributionb,c	Average	27.2213	141.6521	160.8978
	Std Deviation	1.87503	8.50074	9.11882
Most extreme differences	Absolutely	,132	,220	,216
	positive	,132	,214	,216
	negative	-,127	-.220	-.154
Statistics for test		,132	,220	,216
Asymptotic Significance (2-sided)		,200d,e	,188d	,200d,e

a. group_treatment = AF, group_material = 2

b. The distribution to be tested is a normal distribution.

c. Calculated from the data.

i.e. Significance correction according to Lilliefors.

e. This is a lower limit of real significance.

Machine Translated by Google

Kolmogorov-Smirnov test of conformity

		average_removal_ µm	Difference_Ra_n m	Difference_Rq_n m
N		10	10	10
parameters of normal distributionb,c	Average	1.2597	3.2593	4.9424
	Std Deviation	,06135	,40847	,29316
Most extreme differences	Absolutely	,213	,316	,272
	positive	,213	,316	,189
	negative	-,111	-.246	-.272
Statistics for test		,213	,316	,272
Asymptotic Significance (2-sided)		,200d,e	,005d	,035d

a. group_treatment = AF, group_material = 4

b. The distribution to be tested is a normal distribution.

c. Calculated from the data.

i.e. Significance correction according to Lilliefors.

e. This is a lower limit of real significance.

Kolmogorov-Smirnov test of conformity

		average_removal_ µm	Difference_Ra_n m	Difference_Rq_n m
N		10	10	10
parameters of normal distributionb,c	Average	19.0920	10.5451	15.5199
	Std Deviation	1.91656	1.06664	1.97821
Most extreme differences	Absolutely	,227	,148	,265
	positive	,227	,137	,265
	negative	-.160	-.148	-.135
Statistics for test		,227	,148	,265
Asymptotic Significance (2-sided)		,156d	,200d,e	,045d

a. group_treatment = CL, group_material = 1

b. The distribution to be tested is a normal distribution.

c. Calculated from the data.

i.e. Significance correction according to Lilliefors.

e. This is a lower limit of real significance.

Machine Translated by Google

Kolmogorov-Smirnov test of conformity

		average_removal_ µm	Difference_Ra_n m	Difference_Rq_n m
N		10	10	10
parameters of normal distributionb,c	Average	25.4313	47.5482	63.3560
	Std Deviation	2.37003	5.24282	5.28889
Most extreme differences	Absolutely	,275	,229	,227
	positive	,120	,229	,227
	negative	-.275	-.145	-.095
Statistics for test		,275	,229	,227
Asymptotic Significance (2-sided)		,031d	,146d	,155d

a. Group_Treatment = CL, Group_Maerial = 2

b. The distribution to be tested is a normal distribution.

c. Calculated from the data.

i.e. Significance correction according to Lilliefors.

Kolmogorov-Smirnov test of conformity

		average_removal_ µm	Difference_Ra_n m	Difference_Rq_n m
N		10	10	10
parameters of normal distributionb,c	Average	2.4511	5.5013	10.2434
	Std Deviation	,38900	.52991	,62903
Most extreme differences	Absolutely	,241	,121	,220
	positive	,144	.078	,220
	negative	-.241	-,121	-.215
Statistics for test		,241	,121	,220
Asymptotic Significance (2-sided)		,105d	,200d,e	,184d

a. group_treatment = CL, group_material = 3

b. The distribution to be tested is a normal distribution.

c. Calculated from the data.

i.e. Significance correction according to Lilliefors.

e. This is a lower limit of real significance.

Machine Translated by Google

Kolmogorov-Smirnov test of conformity

		average_removal_ µm	Difference_Ra_n m	Difference_Rq_n m
N		10	10	10
parameters of normal distributionb,c	Average	7.0878	14.3827	17.6795
	Std Deviation	,83907	1.57176	2.61959
Most extreme differences	Absolutely	,117	,295	,245
	positive	,103	,295	,245
	negative	-,117	-,137	-.130
Statistics for test		,117	,295	,245
Asymptotic Significance (2-sided)		,200d,e	,014d	,091d

a. Group_Treatment = CL, Group_Maerial = 4

b. The distribution to be tested is a normal distribution.

c. Calculated from the data.

i.e. Significance correction according to Lilliefors.

e. This is a lower limit of real significance.

Kolmogorov-Smirnov test of conformity

		average_removal_ µm	Difference_Ra_n m	Difference_Rq_n m
N		10	10	10
parameters of normal distributionb,c	Average	10.5545	3.8706	5.0639
	Std Deviation	1.09236	,25804	,40636
Most extreme differences	Absolutely	,241	,117	,144
	positive	,178	.095	,124
	negative	-.241	-,117	-.144
Statistics for test		,241	,117	,144
Asymptotic Significance (2-sided)		,104d	,200d,e	,200d,e

a. group_treatment = AF, group_material = KM

b. The distribution to be tested is a normal distribution.

c. Calculated from the data.

i.e. Significance correction according to Lilliefors.

e. This is a lower limit of real significance.

Machine Translated by Google

Kolmogorov-Smirnov test of conformity

		average_removal_µm	Difference_Ra_nm	Difference_Rq_nm
N		10	10	10
parameters of normal distributionb,c	Average	27.2213	141.6521	160.8978
	Std Deviation	1.87503	8.50074	9.11882
Most extreme differences	Absolutely	,132	,220	,216
	positive	,132	,214	,216
	negative	-,127	-.220	-.154
Statistics for test		,132	,220	,216
Asymptotic Significance (2-sided)		,200d,e	,188d	,200d,e

a. Group_Treatment = AF, Group_Maerial = GLZ

b. The distribution to be tested is a normal distribution.

c. Calculated from the data.

i.e. Significance correction according to Lilliefors.

e. This is a lower limit of real significance.

Kolmogorov-Smirnov test of conformity

		average_removal_µm	Difference_Ra_nm	Difference_Rq_nm
N		10	10	10
parameters of normal distributionb,c	Average	,7275	2.0497	4.3007
	Std Deviation	.02187	,20721	,38839
Most extreme differences	Absolutely	,217	,208	,310
	positive	,217	,192	,310
	negative	-.190	-.208	-.179
Statistics for test		,217	,208	,310
Asymptotic Significance (2-sided)		,199d	,200d,e	,007d

a. Group_Treatment = AF, Group_Maerial = Ceramic

b. The distribution to be tested is a normal distribution.

c. Calculated from the data.

i.e. Significance correction according to Lilliefors.

e. This is a lower limit of real significance.

Machine Translated by Google

Kolmogorov-Smirnov test of conformity

		average_removal_ µm	Difference_Ra_n m	Difference_Rq_n m
N		10	10	10
parameters of normal distributionb,c	Average	1.2597	3.2593	4.9424
	Std Deviation	,06135	,40847	,29316
Most extreme differences	Absolutely	,213	,316	,272
	positive	,213	,316	,189
	negative	-,111	-.246	-.272
Statistics for test		,213	,316	,272
Asymptotic Significance (2-sided)		,200d,e	,005d	,035d

a. Group_Treatment = AF, Group_Maerial = GOld

b. The distribution to be tested is a normal distribution.

c. Calculated from the data.

i.e. Significance correction according to Lilliefors.

e. This is a lower limit of real significance.

Kolmogorov-Smirnov test of conformity

		average_removal_ µm	Difference_Ra_n m	Difference_Rq_n m
N		10	10	10
parameters of normal distributionb,c	Average	19.0920	10.5451	15.5199
	Std Deviation	1.91656	1.06664	1.97821
Most extreme differences	Absolutely	,227	,148	,265
	positive	,227	,137	,265
	negative	-.160	-.148	-.135
Statistics for test		,227	,148	,265
Asymptotic Significance (2-sided)		,156d	,200d,e	,045d

a. Group_treatment = CL, Group_material = KM

b. The distribution to be tested is a normal distribution.

c. Calculated from the data.

i.e. Significance correction according to Lilliefors.

e. This is a lower limit of real significance.

Machine Translated by Google

Kolmogorov-Smirnov test of conformity

		average_removal_µm	Difference_Ra_nm	Difference_Rq_nm
N		10	10	10
parameters of normal distributionb,c	Average	25.4313	47.5482	63.3560
	Std Deviation	2.37003	5.24282	5.28889
Most extreme differences	Absolutely	,275	,229	,227
	positive	,120	,229	,227
	negative	-.275	-.145	-.095
Statistics for test		,275	,229	,227
Asymptotic Significance (2-sided)		,031d	,146d	,155d

a. Group_treatment = CL, group_material = GLZ

b. The distribution to be tested is a normal distribution.

c. Calculated from the data.

i.e. Significance correction according to Lilliefors.

Kolmogorov-Smirnov test of conformity

		average_removal_µm	Difference_Ra_nm	Difference_Rq_nm
N		10	10	10
parameters of normal distributionb,c	Average	2.4511	5.5013	10.2434
	Std Deviation	,38900	.52991	,62903
Most extreme differences	Absolutely	,241	,121	,220
	positive	,144	.078	,220
	negative	-.241	-,121	-.215
Statistics for test		,241	,121	,220
Asymptotic Significance (2-sided)		,105d	,200d,e	,184d

a. Group_Treatment = CL, Group_Maerial = Ceramics

b. The distribution to be tested is a normal distribution.

c. Calculated from the data.

i.e. Significance correction according to Lilliefors.

e. This is a lower limit of real significance.

Machine Translated by Google

Kolmogorov-Smirnov test of conformity

		average_removal_ µm	Difference_Ra_n m	Difference_Rq_n m
N		10	10	10
parameters of normal distributionb,c	Average	7.0878	14.3827	17.6795
	Std Deviation	,83907	1.57176	2.61959
Most extreme differences	Absolutely	,117	,295	,245
	positive	,103	,295	,245
	negative	-,117	-,137	-.130
Statistics for test		,117	,295	,245
Asymptotic Significance (2-sided)		,200d,e	,014d	,091d

a. Group_Treatment = CL, Group_Maerial = GOld

b. The distribution to be tested is a normal distribution.

c. Calculated from the data.

i.e. Significance correction according to Lilliefors.

e. This is a lower limit of real significance.

Machine Translated by Google

ranksa

	group_treatment	N	Mean rank	Sum of ranks
mean_removal_µm	AF	10	5.50	55.00
	CL	10	15.50	155.00
	In total	20		
Difference_Ra_nm	AF	10	5.50	55.00
	CL	10	15.50	155.00
	In total	20		
Difference_Rq_nm	AF	10	5.50	55.00
	CL	10	15.50	155.00
	In total	20		

a. Group_Maerial = KM

Statistics for testa,b

	average_removal_µm	Difference_Ra_nm	Difference_Rq_nm
Mann-Whitney-U	,000	,000	,000
Wilcoxon-W	55,000	55,000	55,000
Z	-3,780	-3,780	-3,780
Asymptotic Significance (2-sided)	,000	,000	,000
Exact significance [2*(1-side sig.)]	,000c	,000c	,000c

a. Group_Maerial = KM

b. Group variable: group_treatment

c. Not corrected for bindings.

Machine Translated by Google

ranksa

	group_treatment	N	Mean rank	Sum of ranks
mean_removal_µm	AF	10	12.50	125.00
	CL	10	8.50	85.00
	In total	20		
Difference_Ra_nm	AF	10	15.50	155.00
	CL	10	5.50	55.00
	In total	20		
Difference_Rq_nm	AF	10	15.50	155.00
	CL	10	5.50	55.00
	In total	20		

a. Group_Maerial = GLZ

Statistics for testa,b

	average_removal_µm	Difference_Ra_nm	Difference_Rq_nm
Mann-Whitney-U	30,000	,000	,000
Wilcoxon-W	85,000	55,000	55,000
Z	-1.512	-3,780	-3,780
Asymptotic Significance (2-sided)	,131	,000	,000
Exact significance [2*(1-side sig.)]	,143c	,000c	,000c

a. Group_Maerial = GLZ

b. Group variable: group_treatment

c. Not corrected for bindings.

Machine Translated by Google

ranksa

	group_treatment	N	Mean rank	Sum of ranks
mean_removal_µm	AF	10	5.50	55.00
	CL	10	15.50	155.00
	In total	20		
Difference_Ra_nm	AF	10	5.50	55.00
	CL	10	15.50	155.00
	In total	20		
Difference_Rq_nm	AF	10	5.50	55.00
	CL	10	15.50	155.00
	In total	20		

a. Group_Maerial = ceramics

Statistics for testa,b

	average_removal_µm	Difference_Ra_nm	Difference_Rq_nm
Mann-Whitney-U	,000	,000	,000
Wilcoxon-W	55,000	55,000	55,000
Z	-3,780	-3,780	-3,780
Asymptotic Significance (2-sided)	,000	,000	,000
Exact significance [2*(1-side sig.)]	,000c	,000c	,000c

a. Group_Maerial = ceramics

b. Group variable: group_treatment

c. Not corrected for bindings.

Machine Translated by Google

ranksa

	group_treatment	N	Mean rank	Sum of ranks
mean_removal_µm	AF	10	5.50	55.00
	CL	10	15.50	155.00
	In total	20		
Difference_Ra_nm	AF	10	5.50	55.00
	CL	10	15.50	155.00
	In total	20		
Difference_Rq_nm	AF	10	5.50	55.00
	CL	10	15.50	155.00
	In total	20		

a. Group_Maerial = GOld

Statistics for testa,b

	average_removal_µm	Difference_Ra_nm	Difference_Rq_nm
Mann-Whitney-U	,000	,000	,000
Wilcoxon-W	55,000	55,000	55,000
Z	-3,780	-3,780	-3,780
Asymptotic Significance (2-sided)	,000	,000	,000
Exact significance [2*(1-side sig.)]	,000c	,000c	,000c

a. Group_Maerial = GOld

b. Group variable: group_treatment

c. Not corrected for bindings.

Machine Translated by Google

ranksa

	Group_Maerial	N	medium rank
mean_removal_µm KM		10	25.50
	GLZ	10	35.50
	pottery	10	5.50
	Gold	10	15.50
	In total	40	
Difference_Ra_nm	KM	10	24.40
	GLZ	10	35.50
	pottery	10	5.50
	Gold	10	16.60
	In total	40	
Difference_Rq_nm	KM	10	21:10
	GLZ	10	35.50
	pottery	10	7.40
	Gold	10	18.00
	In total	40	

a. group_treatment = AF

Statistics for testa,b,c

	average_removal_ µm	Difference_Ra_n m	Difference_Rq_n m
Kruskal-Wallis H	36,585	35.153	29,504
df	3	3	3
Asymptotic Significance	,000	,000	,000

a. group_treatment = AF

b. Kruskal-Wallis test

c. Group variable: Group_Maerial

Machine Translated by Google

ranksa

	Group_Maerial	N	medium rank
mean_removal_µm KM		10	25.80
	GLZ	10	35.20
	pottery	10	5.50
	Gold	10	15.50
	In total	40	
Difference_Ra_nm	KM	10	15.60
	GLZ	10	35.50
	pottery	10	5.50
	Gold	10	25.40
	In total	40	
Difference_Rq_nm	KM	10	18.20
	GLZ	10	35.50
	pottery	10	5.50
	Gold	10	22.80
	In total	40	

a. group_treatment = CL

Statistics for testa,b,c

	average_removal_ µm	Difference_Ra_n m	Difference_Rq_n m
Kruskal-Wallis H	36,160	36,440	33,701
df	3	3	3
Asymptotic Significance	,000	,000	,000

a. group_treatment = CL

b. Kruskal-Wallis test

c. Group variable: Group_Maerial

Machine Translated by Google

ranksa

	Group_Maerial	N	Mean rank	Sum of ranks
mean_removal_µm	KM	10	5.50	55.00
	GLZ	10	15.50	155.00
	In total	20		
Difference_Ra_nm	KM	10	5.50	55.00
	GLZ	10	15.50	155.00
	In total	20		
Difference_Rq_nm	KM	10	5.50	55.00
	GLZ	10	15.50	155.00
	In total	20		

a. group_treatment = AF

Statistics for testa,b

	average_removal_µm	Difference_Ra_nm	Difference_Rq_nm
Mann-Whitney-U	,000	,000	,000
Wilcoxon-W	55,000	55,000	55,000
Z	-3,780	-3,780	-3,780
Asymptotic Significance (2-sided)	,000	,000	,000
Exact significance [2*(1-side sig.)]	,000c	,000c	,000c

a. group_treatment = AF

b. Group variable: Group_Maerial

c. Not corrected for bindings.

Machine Translated by Google

ranksa

	Group_Maerial	N	Mean rank	Sum of ranks
mean_removal_µm	KM	10	5.80	58.00
	GLZ	10	15.20	152.00
	In total	20		
Difference_Ra_nm	KM	10	5.50	55.00
	GLZ	10	15.50	155.00
	In total	20		
Difference_Rq_nm	KM	10	5.50	55.00
	GLZ	10	15.50	155.00
	In total	20		

a. group_treatment = CL

Statistics for testa,b

	average_removal_µm	Difference_Ra_nm	Difference_Rq_nm
Mann-Whitney-U	3,000	,000	,000
Wilcoxon-W	58,000	55,000	55,000
Z	-3,553	-3,780	-3,780
Asymptotic Significance (2-sided)	,000	,000	,000
Exact significance [2*(1-side sig.)]	,000c	,000c	,000c

a. group_treatment = CL

b. Group variable: Group_Maerial

c. Not corrected for bindings.

Machine Translated by Google

ranksa

	Group_Maerial	N	Mean rank	Sum of ranks
mean_removal_µm	KM	10	15.50	155.00
	pottery	10	5.50	55.00
	In total	20		
Difference_Ra_nm	KM	10	15.50	155.00
	pottery	10	5.50	55.00
	In total	20		
Difference_Rq_nm	KM	10	14.70	147.00
	pottery	10	6.30	63.00
	In total	20		

a. group_treatment = AF

Statistics for testa,b

	average_removal_µm	Difference_Ra_nm	Difference_Rq_nm
Mann-Whitney-U	,000	,000	8,000
Wilcoxon-W	55,000	55,000	63,000
Z	-3,780	-3,780	-3.175
Asymptotic Significance (2-sided)	,000	,000	.001
Exact significance [2*(1-side sig.)]	,000c	,000c	.001c

a. group_treatment = AF

b. Group variable: Group_Maerial

c. Not corrected for bindings.

Machine Translated by Google

ranksa

	Group_Maerial	N	Mean rank	Sum of ranks
mean_removal_µm	KM	10	15.50	155.00
	pottery	10	5.50	55.00
	In total	20		
Difference_Ra_nm	KM	10	15.50	155.00
	pottery	10	5.50	55.00
	In total	20		
Difference_Rq_nm	KM	10	15.50	155.00
	pottery	10	5.50	55.00
	In total	20		

a. group_treatment = CL

Statistics for testa,b

	average_removal_µm	Difference_Ra_nm	Difference_Rq_nm
Mann-Whitney-U	,000	,000	,000
Wilcoxon-W	55,000	55,000	55,000
Z	-3,780	-3,780	-3,780
Asymptotic Significance (2-sided)	,000	,000	,000
Exact significance [2*(1-side sig.)]	,000c	,000c	,000c

a. group_treatment = CL

b. Group variable: Group_Maerial

c. Not corrected for bindings.

Machine Translated by Google

ranksa

	Group_Maerial	N	Mean rank	Sum of ranks
mean_removal_µm	KM	10	15.50	155.00
	Gold	10	5.50	55.00
	In total	20		
Difference_Ra_nm	KM	10	14.40	144.00
	Gold	10	6.60	66.00
	In total	20		
Difference_Rq_nm	KM	10	11.90	119.00
	Gold	10	9.10	91.00
	In total	20		

a. group_treatment = AF

Statistics for testa,b

	average_removal_µm	Difference_Ra_nm	Difference_Rq_nm
Mann-Whitney-U	,000	11,000	36,000
Wilcoxon-W	55,000	66,000	91,000
Z	-3,780	-2.948	-1.058
Asymptotic Significance (2-sided)	,000	,003	,290
Exact significance [2*(1-side sig.)]	,000c	,002c	,315c

a. group_treatment = AF

b. Group variable: Group_Maerial

c. Not corrected for bindings.

Machine Translated by Google

ranksa

	Group_Maerial	N	Mean rank	Sum of ranks
mean_removal_µm	KM	10	15.50	155.00
	Gold	10	5.50	55.00
	In total	20		
Difference_Ra_nm	KM	10	5.60	56.00
	Gold	10	15.40	154.00
	In total	20		
Difference_Rq_nm	KM	10	8.20	82.00
	Gold	10	12.80	128.00
	In total	20		

a. group_treatment = CL

Statistics for testa,b

	average_removal_µm	Difference_Ra_nm	Difference_Rq_nm
Mann-Whitney-U	,000	1,000	27,000
Wilcoxon-W	55,000	56,000	82,000
Z	-3,780	-3,704	-1,739
Asymptotic Significance (2-sided)	,000	,000	.082
Exact significance [2*(1-side sig.)]	,000c	,000c	,089c

a. group_treatment = CL

b. Group variable: Group_Maerial

c. Not corrected for bindings.

Machine Translated by Google

ranksa

	Group_Maerial	N	Mean rank	Sum of ranks
Average_ablation_µm	GLZ	10	15.50	155.00
	pottery	10	5.50	55.00
	In total	20		
Difference_Ra_nm	GLZ	10	15.50	155.00
	pottery	10	5.50	55.00
	In total	20		
Difference_Rq_nm	GLZ	10	15.50	155.00
	pottery	10	5.50	55.00
	In total	20		

a. group_treatment = AF

Statistics for testa,b

	average_removal_µm	Difference_Ra_nm	Difference_Rq_nm
Mann-Whitney-U	,000	,000	,000
Wilcoxon-W	55,000	55,000	55,000
Z	-3,780	-3,780	-3,780
Asymptotic Significance (2-sided)	,000	,000	,000
Exact significance [2*(1-side sig.)]	,000c	,000c	,000c

a. group_treatment = AF

b. Group variable: Group_Maerial

c. Not corrected for bindings.

Machine Translated by Google

ranksa

	Group_Maerial	N	Mean rank	Sum of ranks
Average_ablation_µm	GLZ	10	15.50	155.00
	pottery	10	5.50	55.00
	In total	20		
Difference_Ra_nm	GLZ	10	15.50	155.00
	pottery	10	5.50	55.00
	In total	20		
Difference_Rq_nm	GLZ	10	15.50	155.00
	pottery	10	5.50	55.00
	In total	20		

a. group_treatment = CL

Statistics for testa,b

	average_removal_µm	Difference_Ra_nm	Difference_Rq_nm
Mann-Whitney-U	,000	,000	,000
Wilcoxon-W	55,000	55,000	55,000
Z	-3,780	-3,780	-3,780
Asymptotic Significance (2-sided)	,000	,000	,000
Exact significance [2*(1-side sig.)]	,000c	,000c	,000c

a. group_treatment = CL

b. Group variable: Group_Maerial

c. Not corrected for bindings.

Machine Translated by Google

ranksa

	Group_Maerial	N	Mean rank	Sum of ranks
Average_ablation_µm	GLZ	10	15.50	155.00
	Gold	10	5.50	55.00
	In total	20		
Difference_Ra_nm	GLZ	10	15.50	155.00
	Gold	10	5.50	55.00
	In total	20		
Difference_Rq_nm	GLZ	10	15.50	155.00
	Gold	10	5.50	55.00
	In total	20		

a. group_treatment = AF

Statistics for testa,b

	average_removal_µm	Difference_Ra_nm	Difference_Rq_nm
Mann-Whitney-U	,000	,000	,000
Wilcoxon-W	55,000	55,000	55,000
Z	-3,780	-3,780	-3,780
Asymptotic Significance (2-sided)	,000	,000	,000
Exact significance [2*(1-side sig.)]	,000c	,000c	,000c

a. group_treatment = AF

b. Group variable: Group_Maerial

c. Not corrected for bindings.

Machine Translated by Google

ranksa

	Group_Maerial	N	Mean rank	Sum of ranks
Average_ablation_µm	GLZ	10	15.50	155.00
	Gold	10	5.50	55.00
	In total	20		
Difference_Ra_nm	GLZ	10	15.50	155.00
	Gold	10	5.50	55.00
	In total	20		
Difference_Rq_nm	GLZ	10	15.50	155.00
	Gold	10	5.50	55.00
	In total	20		

a. group_treatment = CL

Statistics for testa,b

	average_removal_µm	Difference_Ra_nm	Difference_Rq_nm
Mann-Whitney-U	,000	,000	,000
Wilcoxon-W	55,000	55,000	55,000
Z	-3,780	-3,780	-3,780
Asymptotic Significance (2-sided)	,000	,000	,000
Exact significance [2*(1-side sig.)]	,000c	,000c	,000c

a. group_treatment = CL

b. Group variable: Group_Maerial

c. Not corrected for bindings.

Machine Translated by Google

ranksa

	Group_Maerial	N	Mean rank	Sum of ranks
mean_removal_µm	pottery	10	5.50	55.00
	Gold	10	15.50	155.00
	In total	20		
Difference_Ra_nm	pottery	10	5.50	55.00
	Gold	10	15.50	155.00
	In total	20		
Difference_Rq_nm	pottery	10	6.60	66.00
	Gold	10	14.40	144.00
	In total	20		

a. group_treatment = AF

Statistics for testa,b

	average_removal_µm	Difference_Ra_nm	Difference_Rq_nm
Mann-Whitney-U	,000	,000	11,000
Wilcoxon-W	55,000	55,000	66,000
Z	-3,780	-3,780	-2.948
Asymptotic Significance (2-sided)	,000	,000	.003
Exact significance [2*(1-side sig.)]	,000c	,000c	,002c

a. group_treatment = AF

b. Group variable: Group_Maerial

c. Not corrected for bindings.

Machine Translated by Google

ranksa

	Group_Maerial	N	Mean rank	Sum of ranks
mean_removal_µm	pottery	10	5.50	55.00
	Gold	10	15.50	155.00
	In total	20		
Difference_Ra_nm	pottery	10	5.50	55.00
	Gold	10	15.50	155.00
	In total	20		
Difference_Rq_nm	pottery	10	5.50	55.00
	Gold	10	15.50	155.00
	In total	20		

a. group_treatment = CL

Statistics for testa,b

	average_removal_µm	Difference_Ra_nm	Difference_Rq_nm
Mann-Whitney-U	,000	,000	,000
Wilcoxon-W	55,000	55,000	55,000
Z	-3,780	-3,780	-3,780
Asymptotic Significance (2-sided)	,000	,000	,000
Exact significance [2*(1-side sig.)]	,000c	,000c	,000c

a. group_treatment = CL

b. Group variable: Group_Maerial

c. Not corrected for bindings.

Machine Translated by Google

Statisticsa

		average_removal_ µm	Difference_Ra_n m	Difference_Rq_n m
N	Valid	10	10	10
	Missing	0	0	0
Average		10.5545	3.8706	5.0639
median		10.7554	3.8575	5.1075
Std Deviation		1.09236	,25804	,40636
minimum		9.06	3.43	4:31
maximum		11.98	4:21	5.70
25th percentile		9.4400	3.6979	4.8155
	75	11.4768	4.1184	5.3170

a. Group_Maerial = KM, Group_Treatment = AF

Statisticsa

		average_removal_ µm	Difference_Ra_n m	Difference_Rq_n m
N	Valid	10	10	10
	Missing	0	0	0
Average		19.0920	10.5451	15.5199
median		18.7865	10.5444	15.6156
Std Deviation		1.91656	1.06664	1.97821
minimum		15.75	8.81	12.93
maximum		22:41	12.04	18.95
25th percentile		18.2148	9.7863	13.8630
	75	20.3770	11.4466	16.3987

a. Group_Maerial = KM, Group_Treatment = CL

Machine Translated by Google

Statisticsa

		average_removal_µm	Difference_Ra_nm	Difference_Rq_nm
N	Valid	10	10	10
	Missing	0	0	0
Average		27.2213	141.6521	160.8978
median		27.1887	140.5992	161.0108
Std Deviation		1.87503	8.50074	9.11882
minimum		24.34	127.67	146.91
maximum		30.28	156.39	175.69
25th percentile		25.9485	137.7251	154.7457
	75	28.7127	148.2283	165.7204

a. Group_Maerial = GLZ, Group_Treatment = AF

Statisticsa

		average_removal_µm	Difference_Ra_nm	Difference_Rq_nm
N	Valid	10	10	10
	Missing	0	0	0
Average		,7275	2.0497	4.3007
median		,7247	2.0439	4.1896
Std Deviation		.02187	,20721	,38839
minimum		,69	1.66	3.76
maximum		,77	2.30	5.10
25th percentile		,7180	1.9551	4.1258
	75	.7389	2.2814	4.5681

a. Group_Maerial = Ceramics, Group_Treatment = AF

Machine Translated by Google

Statisticsa

		average_removal_ µm	Difference_Ra_n m	Difference_Rq_n m
N	Valid	10	10	10
	Missing	0	0	0
Average		1.2597	3.2593	4.9424
median		1.2478	3.2200	4.9997
Std Deviation		,06135	,40847	,29316
minimum		1:17	2.57	4.39
maximum		1.38	4.01	5.42
25th percentile		1.2232	3.1211	4.7490
	75	1.3021	3.3827	5.0685

a. Group_Maerial = GOld, Group_Treatment = AF

Statisticsa

		average_removal_ µm	Difference_Ra_n m	Difference_Rq_n m
N	Valid	10	10	10
	Missing	0	0	0
Average		7.0878	14.3827	17.6795
median		7.1438	13.9038	16.6783
Std Deviation		,83907	1.57176	2.61959
minimum		5.76	12.01	14.72
maximum		8.28	16.86	22.32
25th percentile		6.3107	13.5309	15.4516
	75	7.7331	16.0984	20.1371

a. Group_Maerial = GOld, Group_Treatment = CL

Machine Translated by Google

Descriptive Statistics

			Statistics	standard error
mean_removal_µm	Average		10.5545	,34544
	95% confidence interval of mean	lower limit	9.7731	
		Upper limit	11.3359	
	5% trimmed mean		10.5586	
	median		10.7554	
	variance		1.193	
	standard deviation		1.09236	
	minimum		9.06	
	maximum		11.98	
	span		2.92	
	interquartile range		2.04	
	crookedness		-.093	,687
	kurtosis		-1,900	1,334
Difference_Ra_nm	Average		3.8706	.08160
	95% confidence interval of mean	lower limit	3.6860	
		Upper limit	4.0552	
	5% trimmed mean		3.8761	
	median		3.8575	
	variance		,067	
	standard deviation		,25804	
	minimum		3.43	
	maximum		4:21	
	span		,78	
	interquartile range		,42	
	crookedness		-.260	,687
	kurtosis		-.616	1,334
Difference_Rq_nm	Average		5.0639	,12850
	95% confidence interval of mean	lower limit	4.7732	
		Upper limit	5.3546	
	5% trimmed mean		5.0707	
	median		5.1075	
	variance		,165	
	standard deviation		,40636	
	minimum		4:31	
	maximum		5.70	

Machine Translated by Google

	span		1.39	
	interquartile range		,50	
	crookedness		-.392	,687
	kurtosis		,297	1,334

a. Group_Maerial = KM, Group_Treatment = AF

Descriptive Statistics

			Statistics	standard error
mean_removal_µm	Average		19.0920	,60607
	95% confidence interval of mean	lower limit	17.7210	
		Upper limit	20.4630	
	5% trimmed mean		19.0928	
	median		18.7865	
	variance		3,673	
	standard deviation		1.91656	
	minimum		15.75	
	maximum		22:41	
	span		6.66	
	interquartile range		2:16	
	crookedness		,298	,687
	kurtosis		,527	1,334
Difference_Ra_nm	Average		10.5451	,33730
	95% confidence interval of mean	lower limit	9.7821	
		Upper limit	11.3081	
	5% trimmed mean		10.5586	
	median		10.5444	
	variance		1.138	
	standard deviation		1.06664	
	minimum		8.81	
	maximum		12.04	
	span		3.23	
	interquartile range		1.66	
	crookedness		-,167	,687
	kurtosis		-.477	1,334
Difference_Rq_nm	Average		15.5199	,62556
	95% confidence interval of mean	lower limit	14.1048	
		Upper limit	16.9351	

Machine Translated by Google

			Statistics	standard error
	5% trimmed mean		15.4731	
	median		15.6156	
	variance		3,913	
	standard deviation		1.97821	
	minimum		12.93	
	maximum		18.95	
	span		6.02	
	interquartile range		2.54	
	crookedness		,611	,687
	kurtosis		-,113	1,334

a. Group_Maerial = KM, Group_Treatment = CL

Descriptive Statistics

			Statistics	standard error
mean_removal_µm	Average		27.2213	,59294
	95% confidence interval of mean	lower limit	25.8799	
		Upper limit	28.5626	
	5% trimmed mean		27.2115	
	median		27.1887	
	variance		3,516	
	standard deviation		1.87503	
	minimum		24.34	
	maximum		30.28	
	span		5.94	
	interquartile range		2.76	
	crookedness		-.017	,687
	kurtosis		-.353	1,334
Difference_Ra_nm	Average		141.6521	2.68817
	95% confidence interval of mean	lower limit	135.5711	
		Upper limit	147.7332	
	5% trimmed mean		141.6099	
	median		140.5992	
	variance		72,263	
	standard deviation		8.50074	
	minimum		127.67	
	maximum		156.39	
	span		28.71	

Machine Translated by Google

	interquartile range		10.50	
	crookedness		.068	,687
	kurtosis		,113	1,334
Difference_Rq_nm	Average		160.8978	2.88362
	95% confidence interval of mean	lower limit	154.3746	
		Upper limit	167.4210	
	5% trimmed mean		160.8535	
	median		161.0108	
	variance		83.153	
	standard deviation		9.11882	
	minimum		146.91	
	maximum		175.69	
	span		28.78	
	interquartile range		10.97	
	crookedness		,263	,687
	kurtosis		-.052	1,334

a. Group_Maerial = GLZ, Group_Treatment = AF

Descriptive Statistics

			Statistics	standard error
mean_removal_µm	Average		25.4313	,74947
	95% confidence interval of mean	lower limit	23.7359	
		Upper limit	27.1267	
	5% trimmed mean		25.5988	
	median		25.3205	
	variance		5,617	
	standard deviation		2.37003	
	minimum		19.63	
	maximum		28.22	
	span		8.60	
	interquartile range		2.28	
	crookedness		-1,682	,687
	kurtosis		4.101	1,334
Difference_Ra_nm	Average		47.5482	1.65793
	95% confidence interval of mean	lower limit	43.7977	
		Upper limit	51.2987	
	5% trimmed mean		47.3182	

Machine Translated by Google

	median		47.0593	
	variance		27,487	
	standard deviation		5.24282	
	minimum		42.00	
	maximum		57.24	
	span		15:23	
	interquartile range		7.68	
	crookedness		,890	,687
	kurtosis		-.027	1,334
Difference_Rq_nm	Average		63.3560	1.67249
	95% confidence interval of mean	lower limit	59.5725	
		Upper limit	67.1394	
	5% trimmed mean		63.1120	
	median		62.6135	
	variance		27,972	
	standard deviation		5.28889	
	minimum		56.41	
	maximum		74.69	
	span		18.28	
	interquartile range		6.66	
	crookedness		1,074	,687
	kurtosis		1,362	1,334

a. Group_Maerial = GLZ, Group_Treatment = CL

Descriptive Statistics

			Statistics	standard error
mean_removal_µm	Average		,7275	.00692
	95% confidence interval of mean	lower limit	,7119	
		Upper limit	,7432	
	5% trimmed mean		,7277	
	median		,7247	
	variance		,000	
	standard deviation		.02187	
	minimum		,69	
	maximum		,77	
	span		,08	
	interquartile range		,02	

Machine Translated by Google

	crookedness		,106	,687
	kurtosis		1,162	1,334
Difference_Ra_nm	Average		2.0497	.06552
	95% confidence interval of mean	lower limit	1.9015	
		Upper limit	2.1980	
	5% trimmed mean		2.0575	
	median		2.0439	
	variance		.043	
	standard deviation		,20721	
	minimum		1.66	
	maximum		2.30	
	span		,64	
	interquartile range		,33	
	crookedness		-.493	,687
	kurtosis		,018	1,334
Difference_Rq_nm	Average		4.3007	,12282
	95% confidence interval of mean	lower limit	4.0229	
		Upper limit	4.5786	
	5% trimmed mean		4.2866	
	median		4.1896	
	variance		,151	
	standard deviation		,38839	
	minimum		3.76	
	maximum		5.10	
	span		1.34	
	interquartile range		,44	
	crookedness		,948	,687
	kurtosis		,942	1,334

a. Group_Maerial = Ceramics, Group_Treatment = AF

Descriptive Statistics

			Statistics	standard error
mean_removal_µm	Average		2.4511	,12301
	95% confidence interval of mean	lower limit	2.1728	
		Upper limit	2.7294	
	5% trimmed mean		2.4585	
	median		2.5026	

Machine Translated by Google

	variance		,151	
	standard deviation		,38900	
	minimum		1.78	
	maximum		2.99	
	span		1:21	
	interquartile range		,53	
	crookedness		-.746	,687
	kurtosis		.078	1,334
Difference_Ra_nm	Average		5.5013	,16757
	95% confidence interval of mean	lower limit	5.1222	
		Upper limit	5.8804	
	5% trimmed mean		5.4989	
	median		5.5239	
	variance		,281	
	standard deviation		.52991	
	minimum		4.64	
	maximum		6.41	
	span		1.77	
	interquartile range		,82	
	crookedness		,002	,687
	kurtosis		-.240	1,334
Difference_Rq_nm	Average		10.2434	,19892
	95% confidence interval of mean	lower limit	9.7934	
		Upper limit	10.6933	
	5% trimmed mean		10.2593	
	median		10.3453	
	variance		,396	
	standard deviation		,62903	
	minimum		9.07	
	maximum		11:13	
	span		2.06	
	interquartile range		,64	
	crookedness		-.518	,687
	kurtosis		,363	1,334

a. Group_Maerial = Ceramics, Group_Treatment = CL

Descriptive Statistics

Machine Translated by Google

			Statistics	standard error
mean_removal_µm	Average		1.2597	,01940
	95% confidence interval of mean	lower limit	1.2158	
		Upper limit	1.3036	
	5% trimmed mean		1.2582	
	median		1.2478	
	variance		,004	
	standard deviation		,06135	
	minimum		1:17	
	maximum		1.38	
	span		,21	
	interquartile range		,08	
	crookedness		,667	,687
	kurtosis		,538	1,334
Difference_Ra_nm	Average		3.2593	,12917
	95% confidence interval of mean	lower limit	2.9671	
		Upper limit	3.5515	
	5% trimmed mean		3.2557	
	median		3.2200	
	variance		,167	
	standard deviation		,40847	
	minimum		2.57	
	maximum		4.01	
	span		1.45	
	interquartile range		,26	
	crookedness		,416	,687
	kurtosis		,855	1,334
Difference_Rq_nm	Average		4.9424	,09271
	95% confidence interval of mean	lower limit	4.7327	
		Upper limit	5.1521	
	5% trimmed mean		4.9466	
	median		4.9997	
	variance		.086	
	standard deviation		,29316	
	minimum		4.39	
	maximum		5.42	
	span		1.03	
	interquartile range		,32	

Machine Translated by Google

	crookedness		-.529	,687
	kurtosis		,602	1,334

a. Group_Maerial = GOld, Group_Treatment = AF

Descriptive Statistics

			Statistics	standard error
mean_removal_µm	Average		7.0878	,26534
	95% confidence interval of mean	lower limit	6.4876	
		Upper limit	7.6881	
	5% trimmed mean		7.0957	
	median		7.1438	
	variance		,704	
	standard deviation		,83907	
	minimum		5.76	
	maximum		8.28	
	span		2.52	
	interquartile range		1.42	
	crookedness		-.052	,687
	kurtosis		-.769	1,334
Difference_Ra_nm	Average		14.3827	,49703
	95% confidence interval of mean	lower limit	13.2583	
		Upper limit	15.5070	
	5% trimmed mean		14.3767	
	median		13.9038	
	variance		2,470	
	standard deviation		1.57176	
	minimum		12.01	
	maximum		16.86	
	span		4.84	
	interquartile range		2.57	
	crookedness		,483	,687
	kurtosis		-.654	1,334
Difference_Rq_nm	Average		17.6795	,82839
	95% confidence interval of mean	lower limit	15.8056	
		Upper limit	19.5535	
	5% trimmed mean		17.5857	
	median		16.6783	

Machine Translated by Google

	variance	6,862	
	standard deviation	2.61959	
	minimum	14.72	
	maximum	22.32	
	span	7.60	
	interquartile range	4.69	
	crookedness	,807	,687
	kurtosis	-.649	1,334

a. Group_Maerial = GOld, Group_Treatment = CL
